# Glycan dysregulation as one of major metabolic subtypes is associated with TERC overexpression and poor outcomes in cervical cancer

**DOI:** 10.3389/fimmu.2025.1585647

**Published:** 2025-08-25

**Authors:** Yanlei Dong, Xinyuan Zhang, Jingjie Zhao, Qingzhen Hou, Yunhai Yu, Yu Wu, Xing Shi, Lina Wang, Dawei Xu

**Affiliations:** ^1^ Gynecology Department, The Second Hospital, Cheeloo College of Medicine, Shandong University, Jinan, China; ^2^ Central Research Laboratory, The Second Hospital, Cheeloo College of Medicine, Shandong University, Jinan, China; ^3^ Department of Biostatistics, School of Public Health, Cheeloo College of Medicine, Shandong University, Shandong, and National Institute of Health Data Science of China, Shandong University, Jinan, China; ^4^ Department of Gynecology and Obstetrics, Liaocheng People’s Hospital, Liaocheng, Shangdong, China; ^5^ Department of Medicine, Division of Hematology, Bioclinicum and Center for Molecular Medicine, Karolinska Institute and Karolinska University Hospital Solna, Stockholm, Sweden

**Keywords:** cervical cancer, glycan dysregulation, metabolic reprogramming, prognostic factor, TERC

## Abstract

**Background:**

Metabolic reprogramming is an important hallmark of cervical cancer (CC), and extensive studies have provided important information for translational and clinical oncology. Here we sought to determine metabolic association with molecular aberrations, telomere maintenance and outcomes in CC.

**Methods:**

RNA sequencing data from TCGA cohort of CC was analyzed for their metabolic gene expression profile and consensus clustering was then performed to classify tumors into different groups/subtypes. The reproducibility of the classification system was further evaluated in GSE68339 CC cohort. The association of metabolic groups with clinical characteristics, telomere maintenance and somatic alterations was assessed to define molecular features of each subtype. Finally, the metabolomic analyses were carried out to directly measure metabolites in tumors and their non-tumorous adjacent tissues (NTs) from 10 CC patients using ultra performance liquid chromatography-mass spectrometry (UPLC-MS).

**Results:**

The analysis of 2752 metabolism-related gene expression in TCGA 304 CC tumors showed a significant expression heterogeneity of these genes. Consensus clustering of these CC tumors identified three distinct metabolic groups (MG), with MG1, MG2 and MG3 characterized by dysregulations in glycans, amino acids/carbohydrates and lipids, respectively. Patients within the MG1 subtype had the shortest disease-free survival (DFS) coupled with robust TERC overexpression. This metabolic stratification was validated in the GSE68339 CC cohort. We further developed a 3 glycan-related gene model (GRGM-3) as a predictor for patient DFS. The TCGA patients were divided into risk-Low and High groups based on their tumor GRGM-3 score using a median cutoff, and those in the risk-High group had significantly shorter DFS. When combined with TERC expression, patients in the high-risk group with high TERC levels had the shortest DFS. Finally, we analyzed metabolites in tumors and NTs from 10 CC patients and further confirmed the metabolic dysregulations identified by gene expression profiling.

**Conclusion:**

Metabolic heterogeneity occurs substantially in CCs and glycan dysregulation is associated with the shortest DFS in CCs. Specifically, the combination of GRGM-3 scores with TERC expression identifies patients with the poorest outcomes, providing a potential tool for individualized risk assessment and contributing to CC precision medicine. It is worth validating our findings for potential clinical application.

## Introduction

Cervical cancer (CC), principally caused by persistent infection of oncogenic human papillomavirus (HPV), is one of the most common female malignancies throughout the world (1–6). Histologically, CC is classified into cervical endocervical adenocarcinoma and squamous cell carcinoma whereas the latter is the predominant CC subtype (2, 3). With the application of Pat tests and HPV vaccination program, the CC incidence and CC-related death have substantially dropped in developed countries, however, such improved scenarios have not occurred significantly in developing countries, and therefore, diagnosis, management and outcome prediction of CCs remain critical issues for woman health (5, 7). Fortunately, recent advances in next-generation sequencing and other high-throughput technologies have drastically facilitated the discovery in the genetic, epigenetic, transcriptomic and proteomic landscapes of CCs (2, 3, 8), which consequently contribute to profound insights into their molecular pathogenesis and biomarker identifications for CC precision medicine. In addition, some new therapeutic strategies have been applied for CC treatment with promising efficacy (9–12).

Unlimited proliferation is one of key cancer hallmarks and maintenance of telomere length is required for such immortal phenotype (13, 14). To achieve this, telomerase reactivation occurs in most cancer types. Telomerase is RNA-dependent DNA polymerase to elongate telomeres and consists of multi components, but its holyenzyme is only composed of telomerase reverse transcriptase (TERT) and telomerase RNA component (TERC) (14). During the CC pathogenesis, the *TERC* gene gain or amplification and upregulation take place early, and even appear in most precursor lesions or cervical intraepithelial neoplasia (CIN), which promotes telomerase activation (4, 15–18). The canonical function of TERC is to serve as a template for telomeric sequence synthesis, but recent studies reveal that it also exhibits telomere lengthening-independent activities (19, 20).

It has also been established that metabolic reprogramming is a key cancer hallmark, which attracts immense interest in cancer metabolism studies (19, 21). Indeed, rapidly dividing cancer cells undergo dramatic alterations in metabolism to ensure efficient nutrient consumption and biomass supplies (21). These metabolic alterations can result from the dysregulation of glucose, lipid and amino acid uptake and consumption, and the application of TCA cycle intermediates in biosynthesis, and among others (21). All the featured changes above also occur in CCs, principally resulting from HPV-induced metabolic alterations directly and indirectly (22, 23). In addition, telomere dysfunction in TERC-knockout mice has been shown to impair mitochondrial biogenesis/function, reduced gluconeogenesis and enhanced reactive oxygen species (11, 24–26). Intriguingly, human TERC RNA can be translated into a protein named hTERP under certain conditions, while hTERP regulates metabolic switch required for cellular hyperproliferation (27). On the other hand, metabolic heterogeneity in CC might be substantial due to differences in infected HPV strains, telomere dysfunction and many other aberrant factors/pathways. Therefore, elucidating such metabolic heterogeneity and identifying metabolism-related biomarkers for CC diagnosis, outcome prediction and therapeutic targets certainly improve CC precision management, which have been explored in the last years (22, 23, 28–30). In the present study, we analyzed metabolism-related gene expression of CC tumors and stratified them into distinct groups or subtypes based on their expression heterogeneity using TCGA (2, 3) and GSE68339 (31) cohorts of CC. By doing so, we identified the glycan dysregulation as one of the major metabolic subtypes in CCs. We further developed a 3 glycan-related gene model (GRGM-3) for disease recurrence and survival prediction. When combined with TERC expression, patients with the poorest outcome were identified. Finally, ultra-performance liquid chromatography (UPLC)-MS-based metabolomic profiling was performed on primary CC samples and matched nontumorous tissues (NTs) to further validate metabolic alterations in CCs.

## Materials and methods

### Patient specimens and UPLC-MS-based metabolomic profiling

Ten CC patients who underwent cervical conization/hysterectomy were recruited, and their clinic-pathological data are summarized in **Supplementary Table S1**. The samples were carefully selected based on specific criteria: Patients with confirmed HPV infection were prioritized, including those with HPV16, HPV18, and a combination of HPV16/12. Additionally, one patient with HPV-negative status was included to provide a broader perspective. All patients had a confirmed pathological diagnosis of either squamous cell carcinoma or adenocarcinoma through histopathological examination. Tumors and their nontumorous cervical tissues were collected and immediately stored at -80°C after surgery. For each sample, 50 mg of tissues were homogenized, centrifuged, and analyzed using UPLC-MS/MS. The study was proved by the Ethical Evaluation Committee of Shandong University Second Hospital (#KYLL-2023LW105). Informed consent was obtained from all the patients.

### Data collection and processing of CC tumors and patient clinical characteristics

The TCGA CC cohort included 304 tumor specimens and 3 non-tumoral counterparts. We collected RNA-seq, mutation, CNV, DNA methylation and clinic-pathological data from the Cancer Genome Atlas (TCGA) Genomic Data commons database (https://gdc.cancer.gov/) (Data were downloaded in Sept. 2021). Patient clinical information is summarized in **Supplementary Table S2**. Microarray-based transcriptomic profiling was performed on 121 CC tumors in the GSE68339 cohort (determined by 4×44K v2 microarray kit), and all data were downloaded from the Gene Expression Omnibus (GEO; https://www.ncbi.nlm.nih.gov/geo/, in Oct. 2021). The patient characteristics in the GSE68339 cohort are listed in **Supplementary Table S3**. For RNA sequencing data, probe-set values were used to determine mRNA abundances after background correction and normalization. The probe values were then mapped to gene symbols, with no additional summarization methods applied.

### Metabolic pathway-based classification of CC

**Figure 1:**
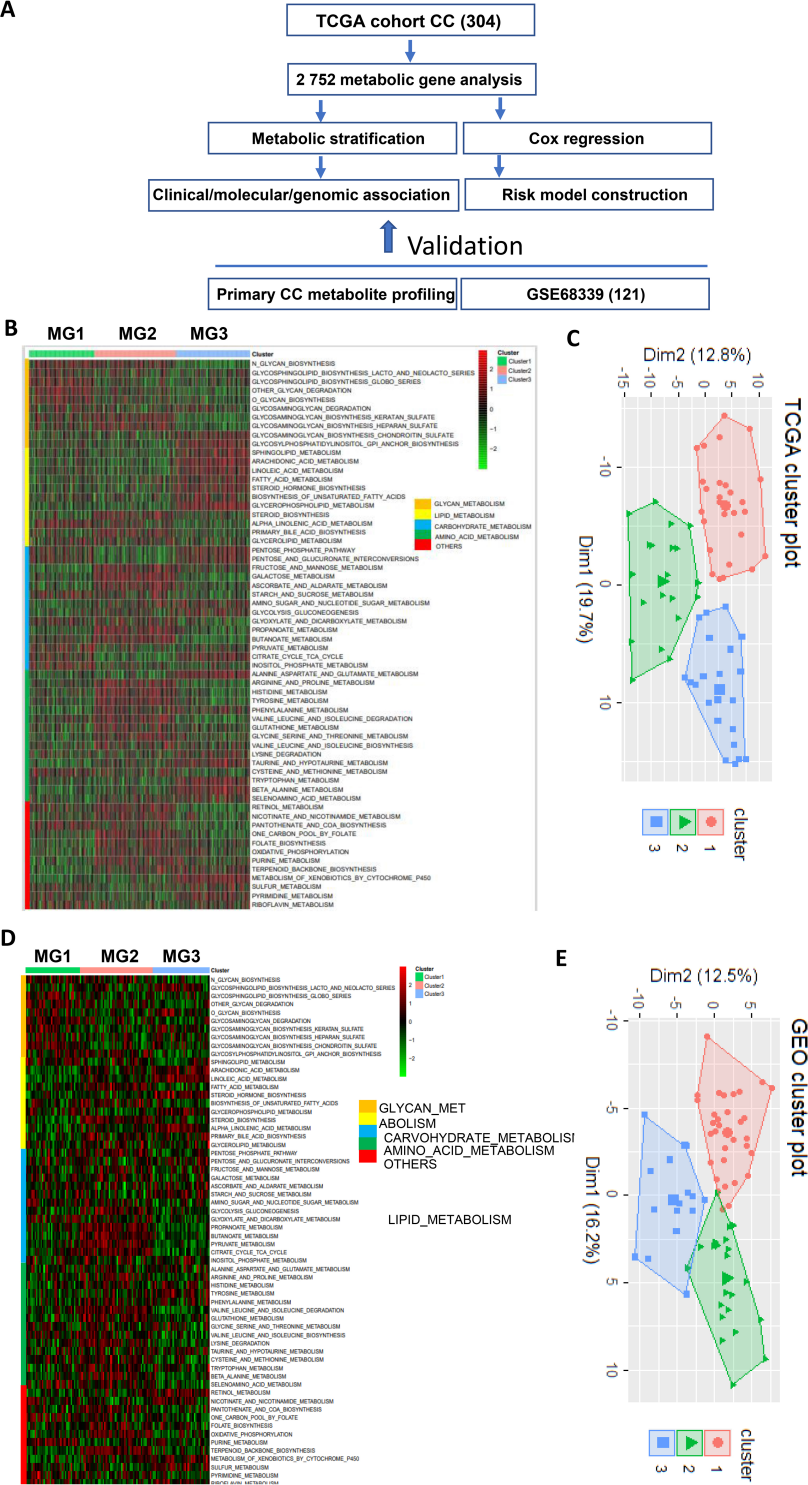
The metabolic classification of cervical cancer (CC). The expression of 2–752 metabolism-related genes was used to classify metabolic subtypes of CC in the TCGA and GSE68339 CC cohorts. **(A)** The flow chart of the study. **(B)** Three metabolic subtypes were identified in the TCGA cohort of CC based on the expression levels of metabolic genes. MG1, MG2 and MG3 were largely characterized by dysregulated glycans, carbohydrate/amino acid and lipids, respectively. **(C)** Principal component analysis (PCA) of metabolism-related gene expression profiles (TCGA cohort). **(D, E)** The analyses of the GSE68339 CC cohort showing similar results.

In the present study, we made the metabolic classification of CC tumors based on the previously identified 2752 metabolism-related genes combined with metabolic pathway analyses (32). The study flow chart was shown in **Figure 1A**. By examining CC tumors in the TCGA and GSE68339 cohorts, we identified that 2585 of these 2752 genes were expressed. Using a GSVA method, we calculated the enrichment score of each metabolic pathway according to the screened metabolic related genes. The obtained metabolic pathway enrichment score was then applied for principal component analysis (PCA) to categorize the CC samples into different metabolic groups. We then adopted Nbclust testing (‘‘NbClust’’ function in R, index = ‘‘all’’) to decide the optimal number of stable metabolic subtypes. (Euclidean distance, k-means clustering from 2 to 10 clusters). The clustering was performed using the GSVA-calculated pathway enrichment scores, rather than the gene expression matrix. Metabolic classification was illustrated using R package “heatmap”.

### Copy number alteration analysis

Somatic CNAs were downloaded from https://xenabrowser.net/. CNA plots were made using R package ‘oncoPrint’ in ‘ComplexHeatmap’.

### The identification of the glycan metabolism-related gene model for disease-free survival prediction

Univariate Cox regression was conducted on glycan gene expression to identify prognostic genes related to DFS in the TCGA CC cohort. The Lasso Cox regression method from the R package “glmnet” was then applied to eliminate collinearity among the prognostic genes. For the risk score calculation, the gene expression values were extracted and normalized. Specifically, the expression values were not standardized, but the raw gene expression data were used in combination with the Lasso-derived coefficients. The risk score was computed as the sum of the selected gene expression values multiplied by their corresponding regression coefficients:

Risk score= coefficients of gene1* expression level of gene1+…+ coefficients of gene n * expression level of gene n.

The risk scores were calculated for each sample, and based on the distribution of these scores, the TCGA CC cohort was divided into high-risk and low-risk groups using the surv_cutpoint method from the “survminer” package. Finally, Kaplan-Meier analysis was performed using the “survival” package based on the risk group classification.

### Time-dependent receiver operating characteristic curves

Time-dependent ROC curves and area under curves (AUCs) were used to estimate the accuracy of identified survival predictors (GRGM-3) in CC patients and made using Rpackage “survivalROC”. The model-based predictive survival time against the observed one was plotted using calibration curves.

### Analyses for differentially expressed genes and pathway enrichment

DEGs (excluding 2585 metabolism-related genes) among different metabolic groups were determined using the “DESeq” package in R software with a cutoff value of *P*<0.05 and log2foldchange>0.5. Kyoto Encyclopedia of Genes and Genomes (KEGG) analyses were performed to explore pathway differences using the R packages “clusterprofile”, “org.Hs.eg.db”, “enrichplot”, and “ggplot2”.

### Epithelial to mesenchymal transition analysis

EMT analysis and score calculation was made based on 77 EMT-related gene expression as described by Mak et al. (33).

### Statistical analysis

We use R software (v4.1.0) to perform all statistical analysis. Comparisons between high and low risk groups and among multi-groups were completed by using Wilcoxon rank test and the Kruskal-Wallis, fisher’s exact test, respectively. A plot of the Kaplan–Meier, multivariate and univariate cox regression analyses were used to evaluate the relationship between the disease-free survival/overall survival and the prognostic gene and clinical feature.

## Results

### Heterogeneity and subtypes of CCs revealed by metabolic gene and pathway analyses

**Figure 1A** shows the flow chart of the present study. A previous study has defined 2752 metabolism-related genes (32), and we first used this panel of genes to comprehensively analyze metabolic alterations in the TCGA cohort of 304 CC patients. A total of 2585 of these 2752 genes were expressed in CC tumors and we thus focused on the expressed genes. A significant heterogeneity of expression in these 2585 genes was observed (**Figure 1B**). By performing consensus clustering on the expression signature of these genes, we were able to stratify CC tumors into three distinct clusters or groups/subtypes, namely MG1 (90/304, 30%), MG2 (112/304, 37%) and MG3 (102/304, 33%). Further metabolic pathway analyses of those heterogeneously expressed genes in each group revealed that MG1, MG2 and MG3 were largely characterized by dysregulated glycans, carbohydrate/amino acid and lipids, respectively. The principal component analysis (PCA) confirmed such differences in expression features within three metabolic groups (**Figure 1C**).

We next evaluated the reproducibility of the metabolic classification obtained from the TCGA CC cohort by analyzing 121 CC tumors in the GSE68339 (31) (**Supplementary Table S3**). The same analysis procedure was carried out and we could categorize them into three metabolic subtypes MG1 (37/121, 31%), MG2 (36/121, 30%) and MG3 (38/121, 31%) (**Figure 1D**). The PCA analysis further shows 3 distinct clusters (**Figure 1E**). Moreover, these 3 subtypes were similarly characterized by dysregulated glycans, carbohydrate/amino acid and lipids, respectively, which was highly consistent with the findings in the TCGA CC cohort.

### Association of the CC metabolic subtypes with clinic-pathological variables

**Figure 2:**
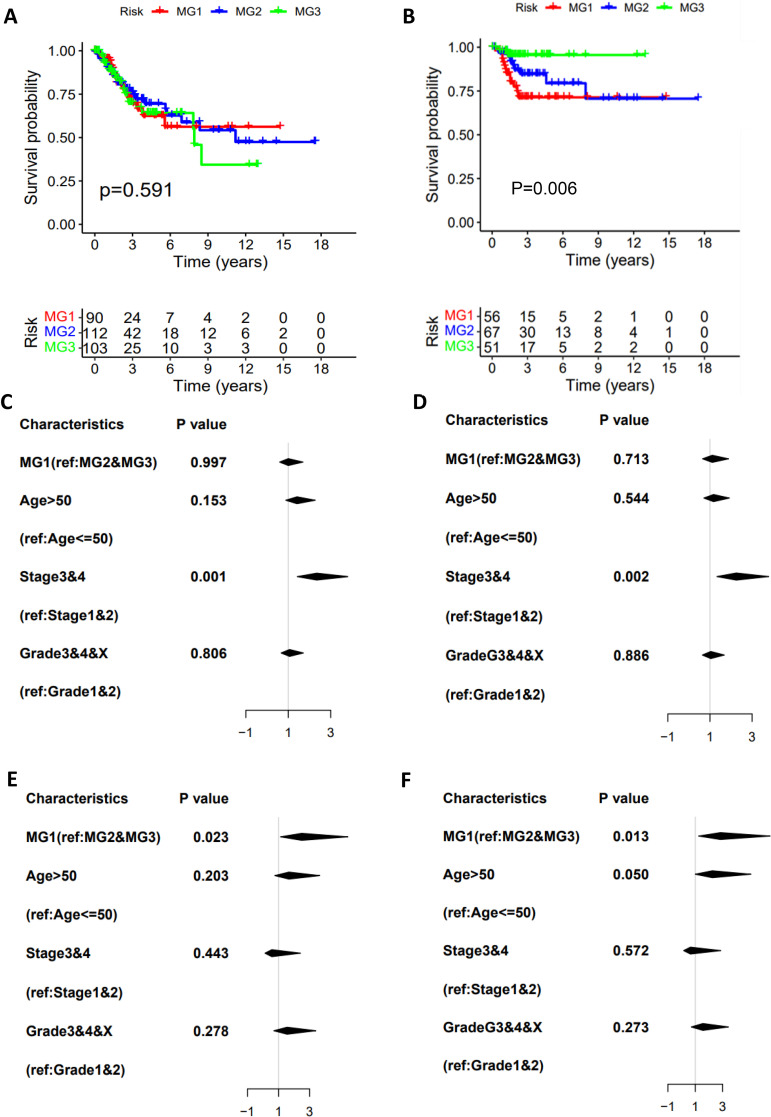
The association of metabolic subtypes with CC patient survival. TCGA CC cohort analysis. The Kaplan–Meier plots show that CC metabolic subtypes are not associated with overall survival (OS) **(A)** but predict disease-free survival (DFS) **(B)**. The univariate **(C)** and multivariate **(D)** COX regression analyses of metabolic subtype association with OS. **(E, F)** The univariate and multivariate COX regression analyses of metabolic subtype association with DFS.

Having identified three metabolic subtypes of CC tumors, we then assessed their association with clinic-pathological variables. In the TCGA cohort, metabolic subtype distributions did not differ between age groups ≤50 and >50 yrs and were not associated with BMI, HPV infection and clinical stages (**Supplementary Table S2**). Adenocarcinoma was more prevalent in MG1 subtype compared to MG2 and MG3. MG1 and MG2 were more frequently observed in tumors at grade III/IV, while lymphovascular invasion and lymph node involvement occurred at higher rates in MG3 (**Supplementary Table S2**). In addition, menopause status was significantly associated with the metabolic subtypes with the highest percentage (60%) of pre-menopause in MG1 patients (**Supplementary Table S2**). We further evaluated whether patient survival was associated with MG subtypes. As shown in **Figure 2A**, there were no differences in overall survival (OS) among three subtypes, but patients in MG1 and MG2 groups had significantly shorter DFS compared with those in the MG3 (**Figure 2B**). Univariate and multivariate cox regression analyses, including the metabolic subtypes, age, grade and stage, showed that only advanced stages were significantly associated with OS (**Figures 2C, D**). The MG1 subtype served as independent prognostic factors for DFS, as assessed by univariate multivariate cox regression analyses (**Figures 2E, F**), whereas age, grade or stage had no effects on patient DFS.

### Association of the CC metabolic subtypes with genomic alterations and DNA methylation

**Figure 3:**
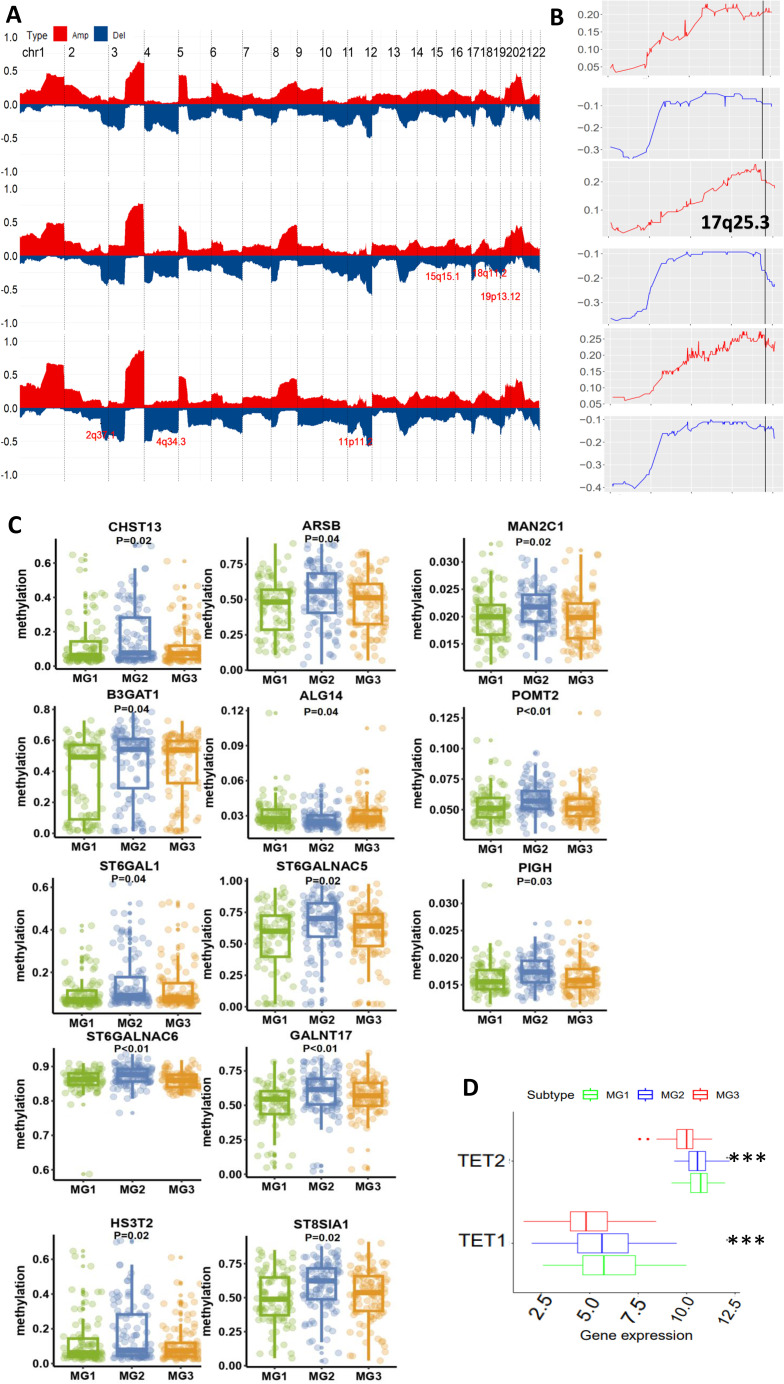
Differential genomic and epigenomic alterations in glycan-related gene loci among three metabolic subtypes (TCGA cohort). **(A)** Global genomic differences among three metabolic CC subtypes. Plots illustrating frequencies of gain/amplification (Red) and deletion (blue) in 22 chromosomes. Some glycan-related gene loci are marked. Top, middle, and bottom: MG1. MG2 and MG3, respectively. **(B)** The detailed analysis of chromosome 17q25.3 where the glycan-related genes are located. **(C)** Differences in DNA methylation levels of 13 glycan-related genes among three metabolic CC subtypes. **(D)** TET1 and TET2 expression among three metabolic CC subtypes. ***: P < 0.001.

The results above unravel that patients in the MG1 subtype featured with glycan dysregulation, exhibited the worst outcomes. To determine underlying mechanisms, we first analyzed genomic and epigenomic factors that contribute to enhanced glycan expression. We compared the alterations in the loci associated with the glycan metabolism and observed no differences in their amplification among 3 subtypes (**Figure 3A**). However, 17q25.3, associated with glycan metabolism, was deleted with much higher frequencies in both MG2 and MG3 subtypes (**Figure 3B**). The loss of other loci involved in the glycan metabolism also occurred specifically in MG2 and MG3, which included 15q15.1 (CHST14), and 5q35.3 (MGAT1, MGAT4B and B4GALNT7) in MG2, while 1p36.11 (DDOST, EXTL1, FUCA1, PIGV, MAN1C1 and B3GALT6), 2q37.1 (NEU2 and B3GNT7), 7q36.1 (SPAM1, HYAL4, CHPF4, GALNT11 and GALNTL5) and 11p11.2 (EXT2 and CHST1) in MG3 (**Figures 3A, B**).

As aberrant DNA methylation is widespread in CCs (31), we examined whether this is also a driving-force for glycan dysregulation in MG1 tumors. Our analyses revealed that 13 glycan metabolism-related genes (ALG14 (1 p21.3), ARSB (5q14.1), B3GAT1 (11q25), GALNT17 (7q11.22), CHST13 (3q21.3), SH3ST2, MAN2C1 (15q24.2), PIGH (14q24.1), POMT2 (14q24.3), ST6GALNAC5 (1p31.1), ST6GAL1 (3q27.3), ST6GALNAC6 (9q34.11), and ST8SIA1 (12p.12.1)) had the lowest methylation levels in MG1 compared to MG2 and MG3 subtypes (**Figure 3C**). Moreover, a significantly inverse correlation between expression and methylation levels of these genes was documented (**Supplementary Figure S1**). Because ten eleven translocation (TET) family enzymes oxidize 5-methylcytosines (5mCs) and induce locus-specific reversal of DNA methylation, we further compared expression differences in the *TET1* and *TET2* genes (TET3 data unavailable) among three subtypes. Expression levels of both TET1 and TET2 were highest in the MG1 subtype (**Figure 3D**).

### Differences in oncogenic pathways, epithelial-mesenchymal transition, and TERC expression among three metabolic groups of CC

**Figure 4:**
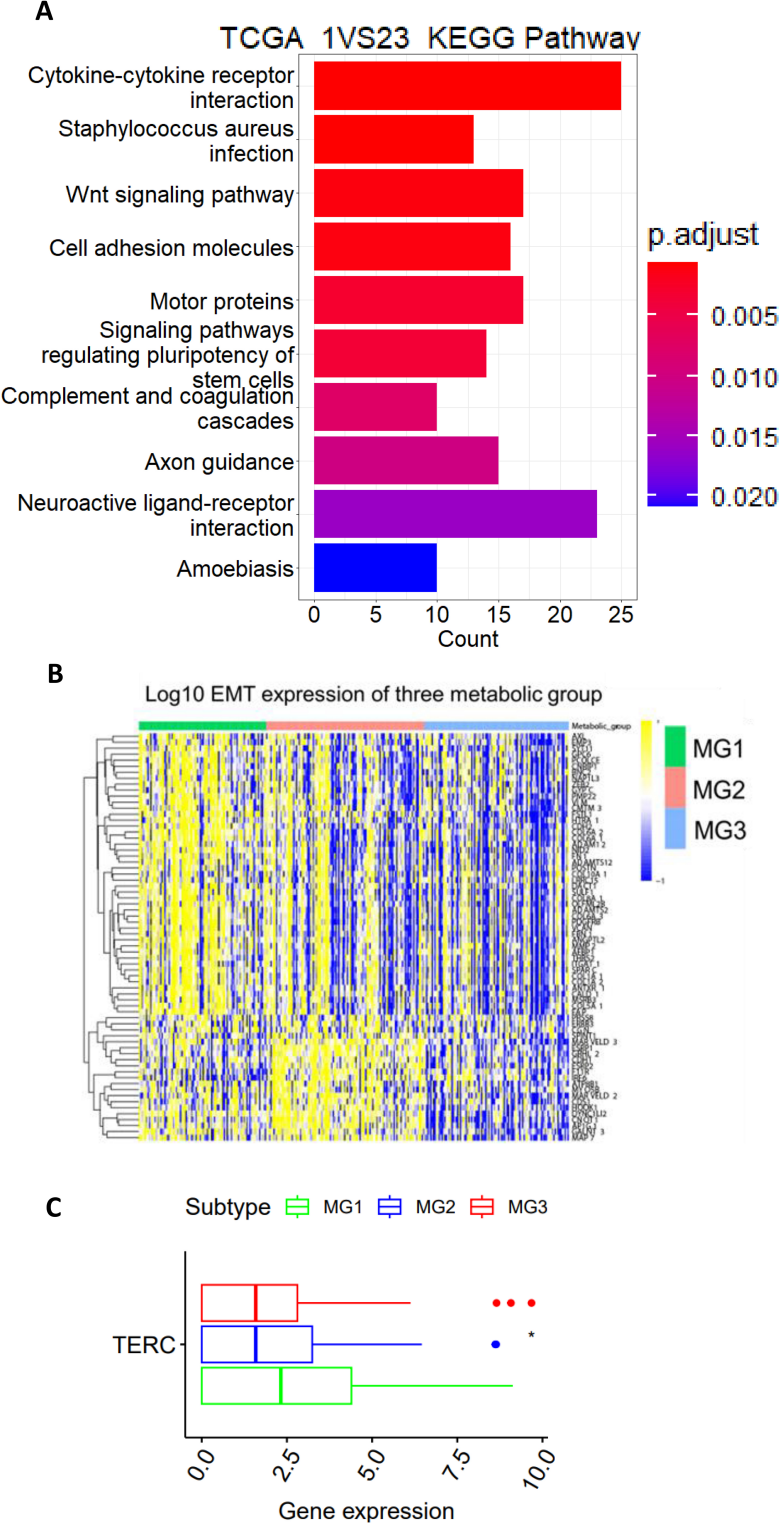
The TCGA CC MG1 subtype is characterized by the aggressive phenotype and TERC overexpression. **(A)** The KEGG analysis showing the significantly enrichment of wnt, stem cell and PIK3CA-AKT pathways in MG1 tumors. **(B)** Significantly enhanced expression of EMT genes in MG1 tumors. **(C)** TERC expression among three metabolic CC subtypes. *: P < 0.05.

Given the shortest DFS in the MG1 subtype, we sought to probe the underlying mechanisms. The KEGG analysis was first performed to identify featured signaling pathways in MG1 tumors. The most enriched pathways include cytokine-cytokine receptor interaction, WNT, cell adhesion, signaling pathway regulating pluripotent stem cells, PI3K-AKT, ECM receptor interaction and melanoma (**Figure 4A**).

All these pathways are known to drive cancer aggressiveness, and because most of them induce EMT through which disease progression or metastasis occurs, we compared its difference among 3 CC subtypes. The EMT signature score was calculated as previously described (33), and as expected, the MG1 subtype had the highest EMT score (**Figure 4B**). In addition, the *TERC* gene at 3q26 is one of the early altered genes in the pathogenesis of CC and its overexpression leads to telomerase activation required for malignant transformation and/or progression (16, 17). We further analyzed TERC expression, and its level was highest in MG1 (**Figure 4C**).

### A 3 glycan-related gene signature for DFS prediction in CC patients

**Figure 5:**
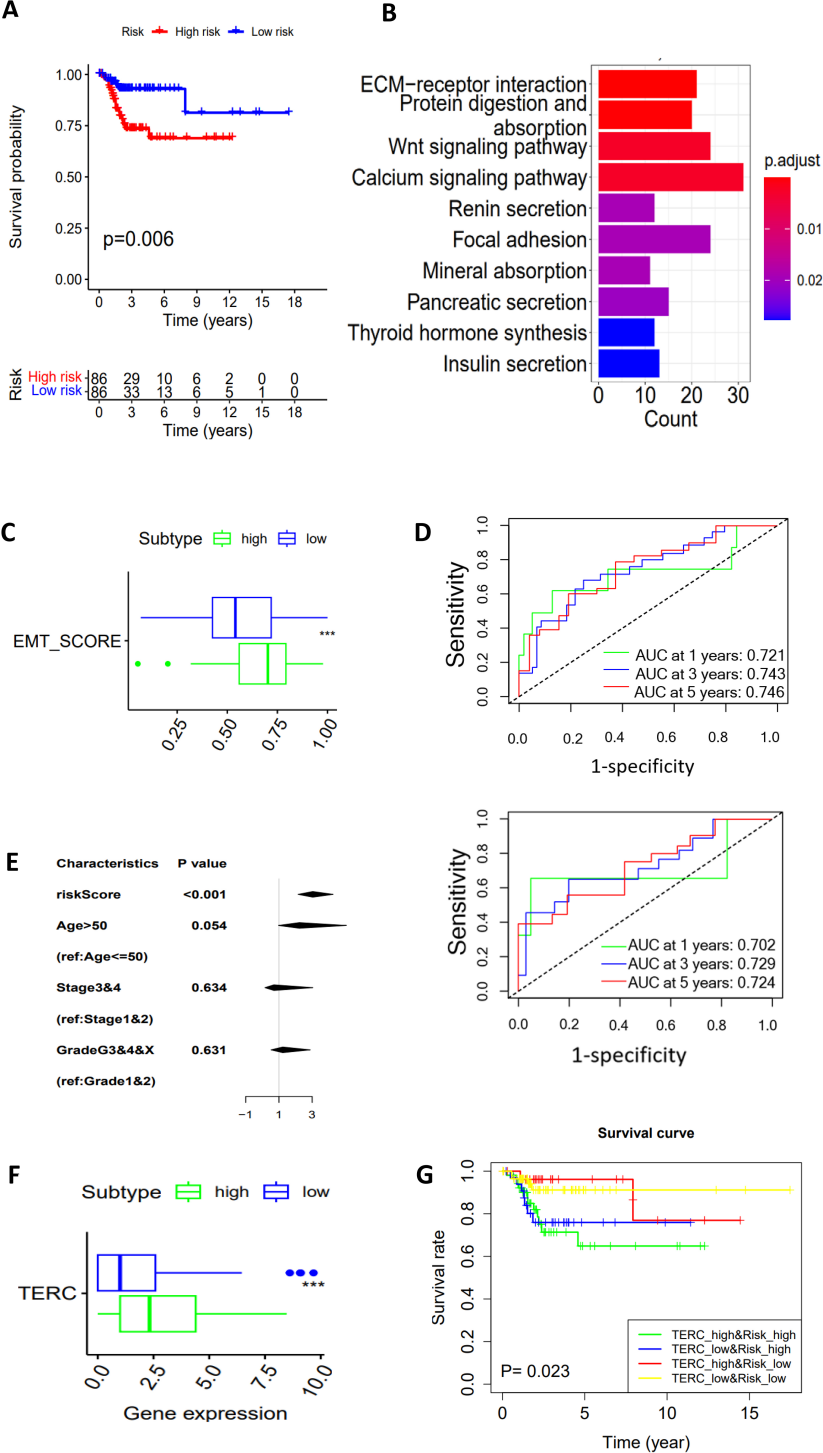
A 3 glycan-related gene model (GRGM-3) as a prognostic factor for DFS in the TCGA cohort of CC patients. **(A)** Patients within the risk high group are associated with disease-free survival (DFS) as shown by the Kaplan–Meier plot. Patients were categorized into high- and low-score groups using the median value as the cutoff. **(B)** The enriched pathways in the risk-high tumors as determined by KEGG analysis. **(C)** Higher EMT scores in the risk-high tumors. **(D)** Time-dependent ROC curves and area under curves (AUCs) for the accuracy estimation of the risk score for recurrence. In the top panel, all the patients were included for analyses, and bottom panel: Only stage I patients. **(E)** The multivariate COX regression analyses of GRGM-3 score association with DFS. The median score was used for cutoff. **(F)** Differences in TERC expression between GRGM-3 high- and low-groups. **(G)** Higher TERC expression combined with the higher GRGM-3 score predicts patients with the worst outcomes. ***: P < 0.001.

The results above showed that CC metabolic subtypes were significantly associated with DFS, indicating a clinical significance. However, such analyses need special bioinformatic knowledge, and are time-consuming and cost unfriendly. We thus sought to construct a simple glycan-related gene model or GRGM-3 suitable for clinical routine application. To this end, we performed Lasso Cox regression analysis and eventually identified a panel of 3 glycan metabolism-related genes (GALNT16, PIGT and GALNT15) (Risk Score = 0.000257 GALNT16 + 0.00003 PIGT + 0.000992 GALNT15). Based on the expression of these 3 genes, we calculated their GRGM-3 risk score in each CC tumor and then divided them into Risk-Low and High groups using a median value as a cut-off point. The Kaplan-Meier analysis revealed that patients in the risk-low group exhibited significantly longer DFS (**Figure 5A**). The KEGG analysis revealed the enriched WNT, ECM receptor interaction, cell adhesion and other signaling pathways in the GRGM-3 high risk group (**Figure 5B**) which was coupled with a significantly higher EMT score (**Figure 5C**). We further evaluated the accuracy of the GRGM-3 for recurrence prediction. First, we included patients at all the stages, the model displayed a high accuracy in predicting 1-, 3- and 5-year DFS with area under curve (AUC) > 0.72, as assessed by a time-dependent ROC curve (**Figure 5D** top). One hundred and six patients at stage I were then assessed separately, and similar results were obtained (**Figure 5D** bottom). Multivariate COX regression analyses were further carried out by including the GRGM-3, age (≤50 and >50 yrs), stages and grades, and the GRGM-3 was the only independent prognostic variable for DFS (**Figure 5E**). TERC expression was robustly higher in the GRGM-3-high tumors (**Figure 5F**). When the GRGM-3 was combined with TERC expression, we observed that patients within the risk-high group accompanied by higher TERC expression exhibited the shortest DFS (**Figure 5G**).

### The CC metabolic alterations by directly analyzing metabolites in primary CC tumors

**Figure 6:**
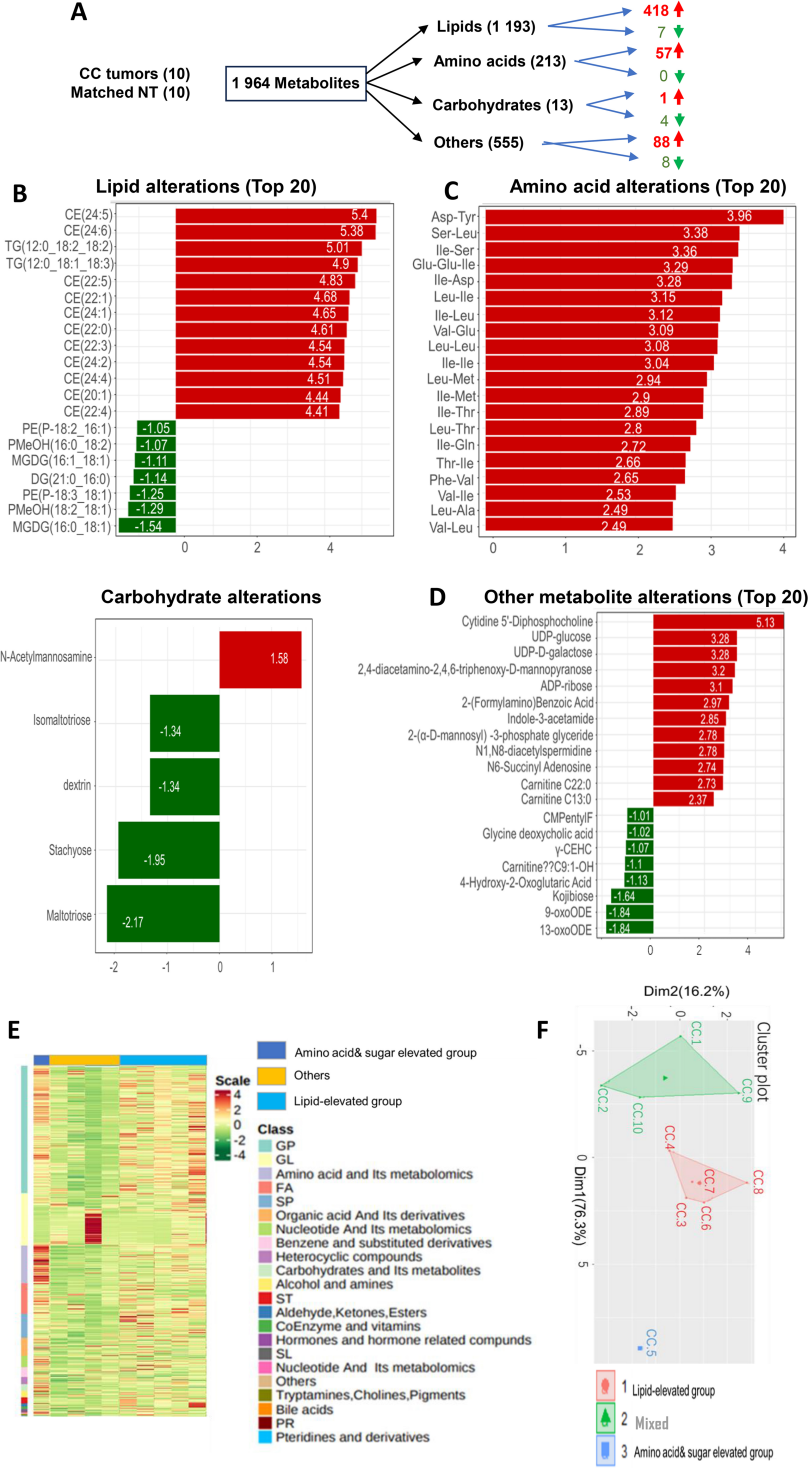
Alterations in metabolites and metabolite-based grouping of primary CC tumors. Ultra-performance liquid chromatography (UPLC)-MS-based metabolomic profiling was performed on primary CC samples and matched nontumorous tissues (NTs) from 10 CC patients. **(A)** A total of metabolites analyzed and up- and down-regulated products. **(B-D)** The top altered metabolites (Lipids, amino acids, carbohydrates, and others) in CC tumors compared to NTs. **(E)** The heatmap showing metabolite differences in 10 tumors. **(F)** Principal component analysis (PCA) of metabolite profiles.

Finally, to assess whether the transcriptome-based metabolic classification is valid, we directly determined abundances of metabolites in primary CC tumors (**Supplementary Table S1**). A total of 1–964 metabolites were assessed and significant differences were observed between adjacent NTs and tumors (**Figure 6A**). In all 1–193 lipids analyzed, 418 of them increased while only 7 decreased in CC tumors; 4 of 13 carbohydrates were downregulated whereas 1 was up-regulated; the significantly increased level of amino acids occurred in 57 of 213 and none of them decreased; among 555 other metabolites, including cytidine-5′-diphosphocholine, UDP-glucose, etc., 88 and 8 of them had increased and reduced levels in tumor tissues, respectively (**Figure 6A**). The top 20 altered metabolites were further shown in **Figures 6B–D**. Based on all the results above, tumors could be tentatively grouped into the following metabolic groups: Lipid (5 cases) and amino acid/carbohydrate (4 cases) subgroups, while 1 tumor was mixed with multiple metabolite dysregulations (**Figures 6E, F**). Consistently, the PCA analysis shows 3 different clusters of 10 tumors (**Figure 6F**).

## Discussion

In the present study, we comprehensively investigated metabolic dysregulation in CCs and underlying molecular/clinical implications. By analyzing the TCGA and GSE68339 cohorts of CCs, we classified CC tumors into three distinct metabolic subtypes, and each of them was associated with different metabolic pathways, molecular and clinical features and different outcomes. We further confirmed gene expression-based metabolic stratification via a direct assessment of metabolites in primary CC tumors. Moreover, a 3 glycan-related gene risk model (GRGM-3) was developed as an independent prognostic factor for DFS in CCs.

A previous study analyzed metabolic alterations in the TCGA cohort of CCs and classified tumors into three metabolic subtypes, too (30), however, it was unclear about the featured metabolic alterations in each subtype. In the present study, we identified three distinct metabolic subtypes of CC tumors that were characterized by dysregulations in glycans, amino acids/carbohydrates and lipids, respectively. Glycans has long been recognized to participate in oncogenesis and to play important parts in cell adhesion, cell-cell communication, angiogenesis, stemness, invasion, metastasis, and immunological regulation (34–38), but there have been no reports so far to show their dysregulation as a major type of metabolic reprograming in cancer. Our findings further unraveled that patients in the glycan subtype (MG1) had the shortest DFS compared to two other subtypes. KEGG analysis showed the enriched WNT pathway in this subtype, consistent with the enhanced activity of WNT proteins upon their glycosylation (22, 34). Glycans regulate stemness by binding to WNT, and other signaling ligands (22, 34). However, we did not observe a higher stem cell score in the MG1 subtype. Instead, the MG1 tumors exhibited the highest EMT score, which is expected because the WNT signaling also promotes EMT. In addition, the extracellular matrix (ECM) receptor interaction pathway is significantly enriched in the MG1 subtype. ECM plays a key role in EMT induction and may thus be another driving-force to enhance EMT. Consistently, the enriched WNT and ECM pathways coupled with higher EMT scores were observed in the GRGM high-risk tumors.

The aberrant expression of glycan-related genes has been observed from precursor lesions to metastatic CC (36, 37, 39–41). Thus, we further explored mechanisms underlying the dysregulated glycan metabolism in CC tumors. First, in MG2 and MG3 subtype tumors, we observed the loss of several loci where key glycan-metabolic genes are localized, which may compromise glycan metabolism in those tumors. Second, compared to MG2 and MG3 tumors, the MG1 subtype exhibited the lowest levels of DNA methylation in 13 glycan-related genes, and in all of them, there was a significantly inverse correlation between their expression and methylation levels. It is well characterized that several oncometabolites contribute to aberrant DNA methylation in renal cell carcinoma (42). However, it is currently unclear whether this is also the case in CC tumors. Nevertheless, Vojta et al. showed that expression of glycan-related genes was regulated by DNA methylation (43). Consistently, we observed the highest levels of TET1 and TET2 expression in the MG1 group, and the hypomethylation of the glycan-metabolic genes might play an important part in their upregulation in these tumors. In addition, the HPV-derived E6 onco-protein has been shown to promote glycosyltransferase ST6GAL1 expression (44) and HPV infection may result in dysregulation of glycan- and other metabolites-related genes more broadly (35, 36). Collectively, multi-mechanisms are attributable to perturbations of glycan metabolism-related genes in CCs. It should also be pointed out that confounding clinical variables or co-occurring genetic alterations and other factors may play a part in the observed difference in metabolism associated with patient outcomes.

Metabolic stratification of CCs based on 2752 metabolism-related genes is time-consuming and costly, and need special bioinformatic knowledge, which is difficult to be directly applied in clinical routine. To solve these problems, we further developed a 3 glycan-related gene risk model (GRGM-3) to predict patient survival. The model displayed a high accuracy in predicting 1-, 3- and 5-year DFS. Interestingly, high expression of GALNT15, one of 3 genes in GRGM-3, was recently shown to predict poor outcomes in gastric cancer patients, and associated with pro-tumorous immune cell infiltration (45). These and other findings support that glycan dysregulation contributes to immunosuppression, and cancer progression (8, 9, 45). However, it is currently unclear how GALNT15 or other glycan-related factors affect tumor immune-microenvironment, and whether they can serve as biomarkers to predict patient response to immunotherapy. Further investigations are required to elucidate these issues.

We further performed UPLC-MS to directly assess the metabolite alterations in CC tumors. Our findings revealed that lipid and amino acid/carbohydrate dysregulation occurred in 5 and 4 tumors, respectively, whereas one was mixed with dysregulations in different types of metabolites. Because the sample size is small, the analysis result does not exactly mimic the metabolic subtypes classified in the TCGA CC cohort. Nevertheless, it is evident from the present study that mRNA-based CC metabolic classification is largely consistent with the data obtained from metabolite assessments.

TERC, as one of the key components in the telomerase complex, plays an important role in telomere stabilization during the CC pathogenesis (14, 17). Interestingly, both *TERC* and glycan-related genes already alter in CIN (17, 35, 36), and the GRGM-3 risk-high tumors express significantly higher levels of TERC RNA. Although TERC expression itself is not associated with patient survival, when combined with the GRGM-3 score, we observed that patients within the risk-high group accompanied by higher TERC expression had the shortest DFS. Thus, this combination helps identify patients with the poorest outcome who need close follow-up and care.

Our study has limitations. First, metabolomic and transcriptomic data from datasets are analyzed. Second, the CC cohort for metabolite assessment is small, which constrains metabolic profiling and analyses in CC patients. Therefore, it is important to validate our findings experimentally and clinically by recruiting more CC patients.

## Conclusions

By analyzing metabolism-related gene expression of CC tumors in the TCGA and GSE68339 cohorts, we categorized them into 3 distinct subtypes, and identified the glycan dysregulation as one of the major metabolic subtypes in CCs. We further developed a 3-gene signature for disease recurrence and survival prediction. When combined with TERC expression, patients with the poorest outcome were stratified. Direct metabolite profiling of primary CC tumors further validates metabolic alterations in CCs. The present findings contribute to better understanding of metabolic reprograming, and reveal its crosstalk with telomere maintenance mechanisms, and clinical implications in CC. It should be pointed out, however, that validation of our results experimentally and clinically is required to make a solid conclusion.

## Data Availability

The datasets presented in this study can be found in online repositories. The names of the repository/repositories and accession number(s) can be found in the article/**Supplementary Material**.
