# Supplementary material for: Glycan dysregulation as one of major metabolic subtypes is associated with TERC overexpression and poor outcomes in cervical cancer
Source: Front Immunol. 2025 Aug 25;16:1585647. doi: 10.3389/fimmu.2025.1585647 (PMC12414962; doi:10.3389/fimmu.2025.1585647)

Figure S1. The inverse correlation between expression and DNA methylation in the key glycan genes. The TCGA CC cohort of 304 tumors were analyzed for the correlation between expression and DNA methylation in 13 key glycan genes (downloaded from CiBioportal).

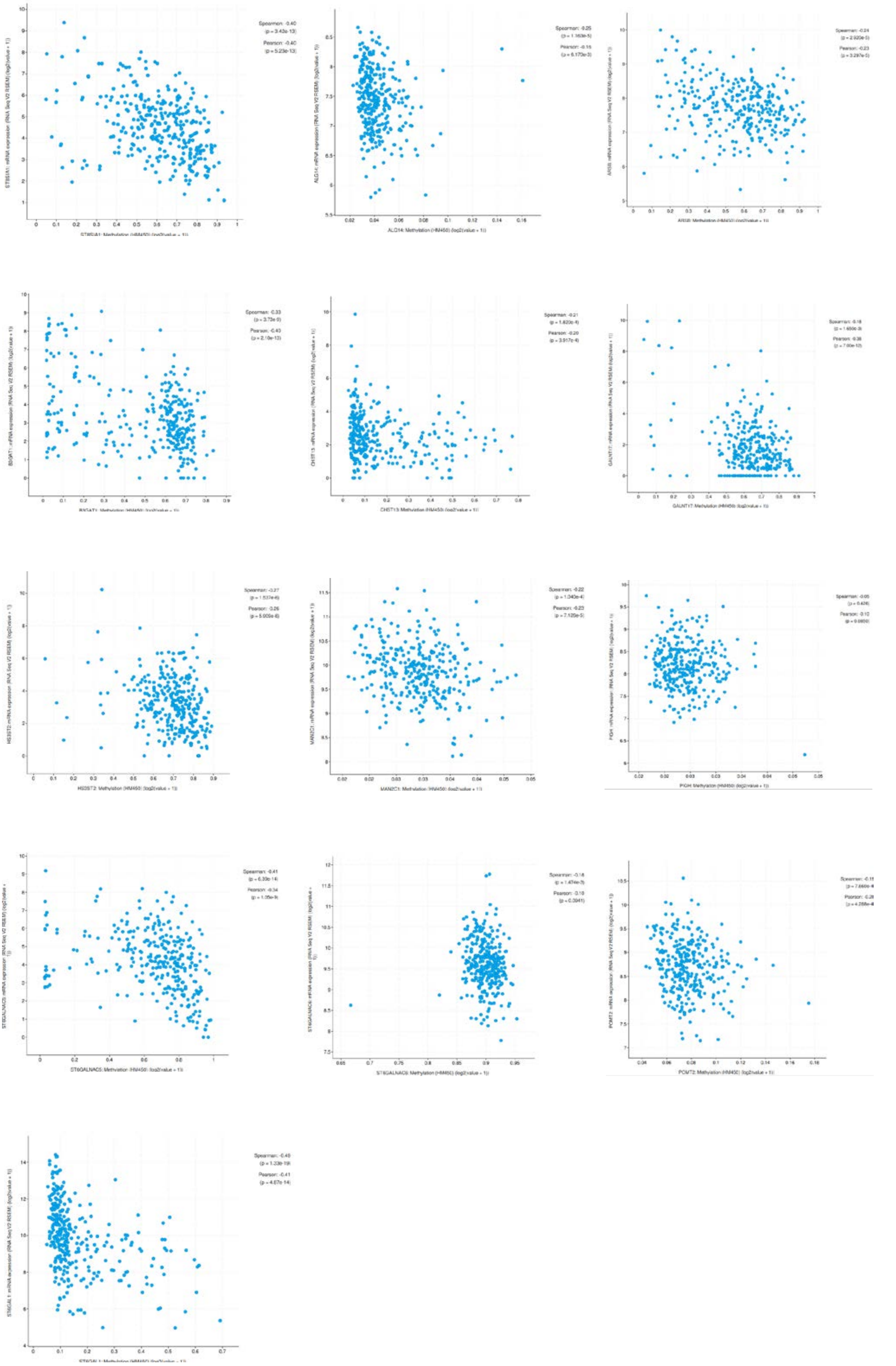

Supplement: Supplementary file 1 [file DataSheet1.zip › Supplementary_Material_Presentation/Fig. S1.pdf]
