# Supplementary figures and images for: Glycan dysregulation as one of major metabolic subtypes is associated with TERC overexpression and poor outcomes in cervical cancer
Source: Front Immunol. 2025 Aug 25;16:1585647. doi: 10.3389/fimmu.2025.1585647 (PMC12414962; doi:10.3389/fimmu.2025.1585647)

A

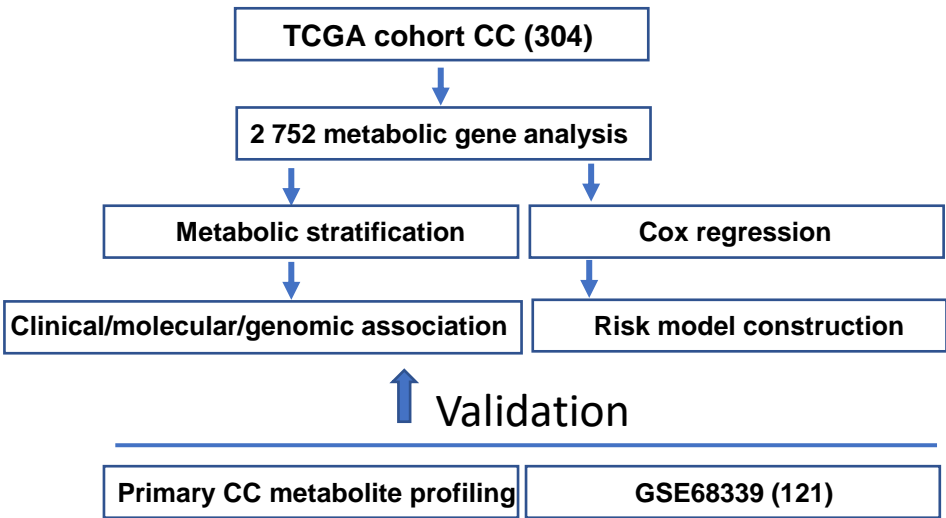

B

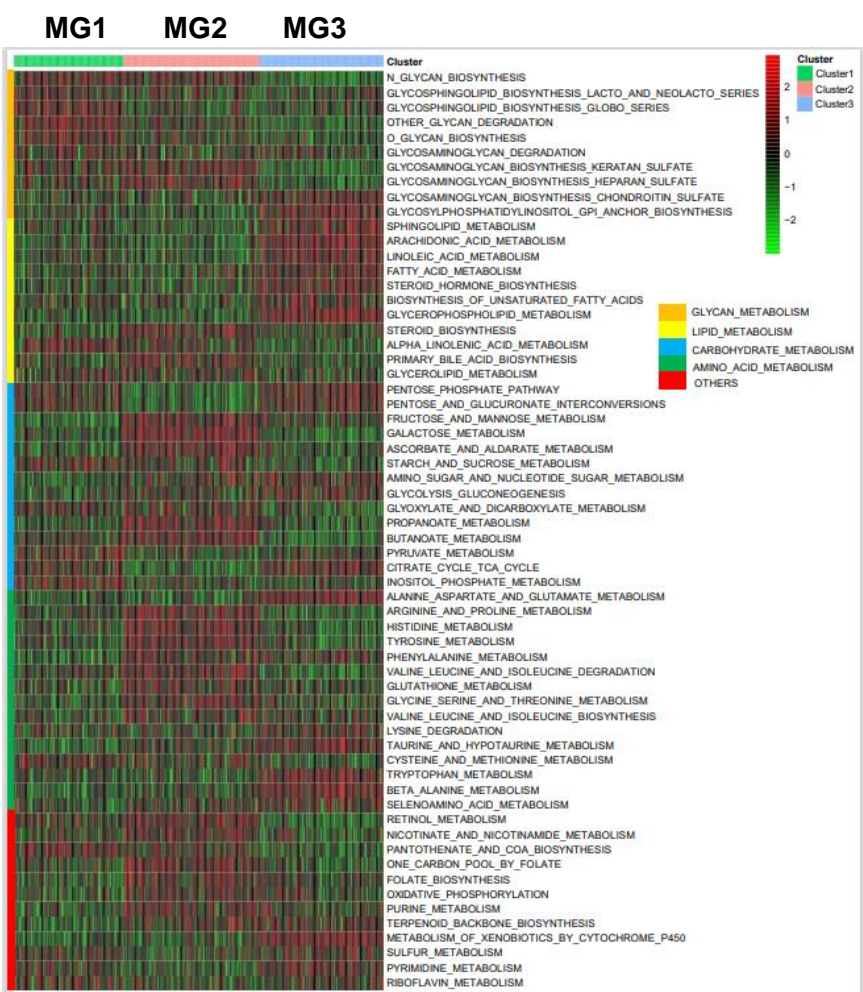

C

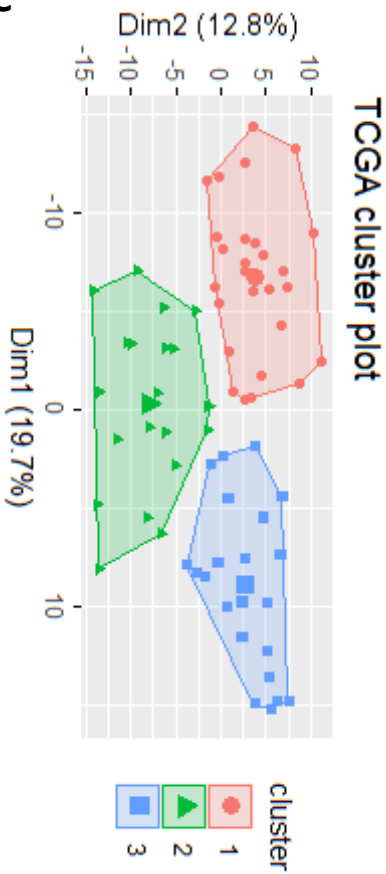

D

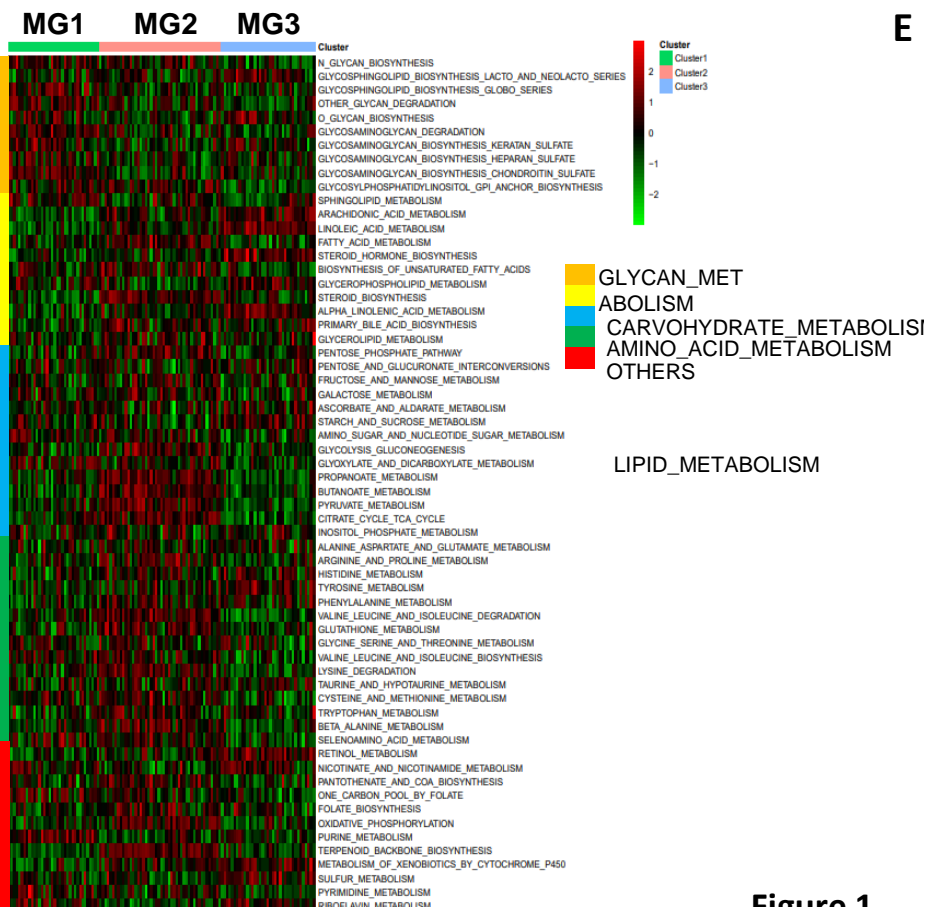

E

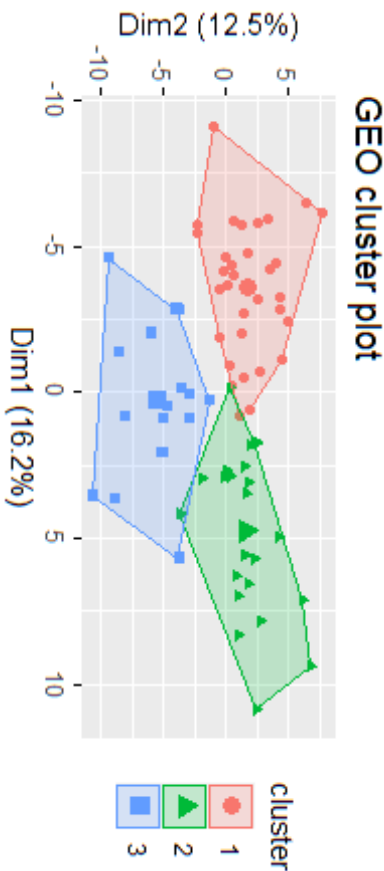

Figure 1

Supplement: Supplementary file 1 [file DataSheet1.zip › Supplementary_Material_Presentation/Fig 1.pdf]

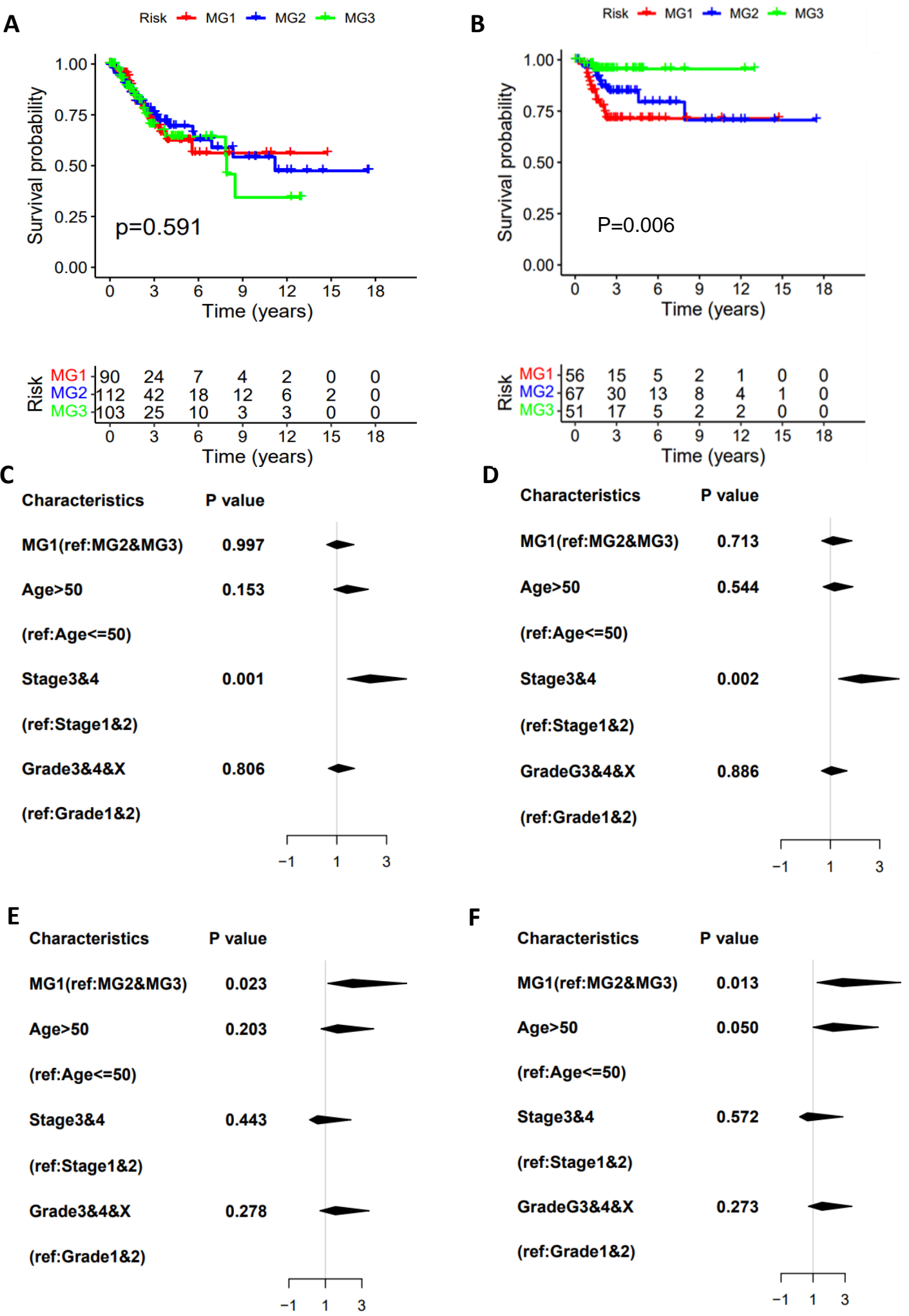

Supplement: Supplementary file 1 [file DataSheet1.zip › Supplementary_Material_Presentation/Fig 2.pdf]

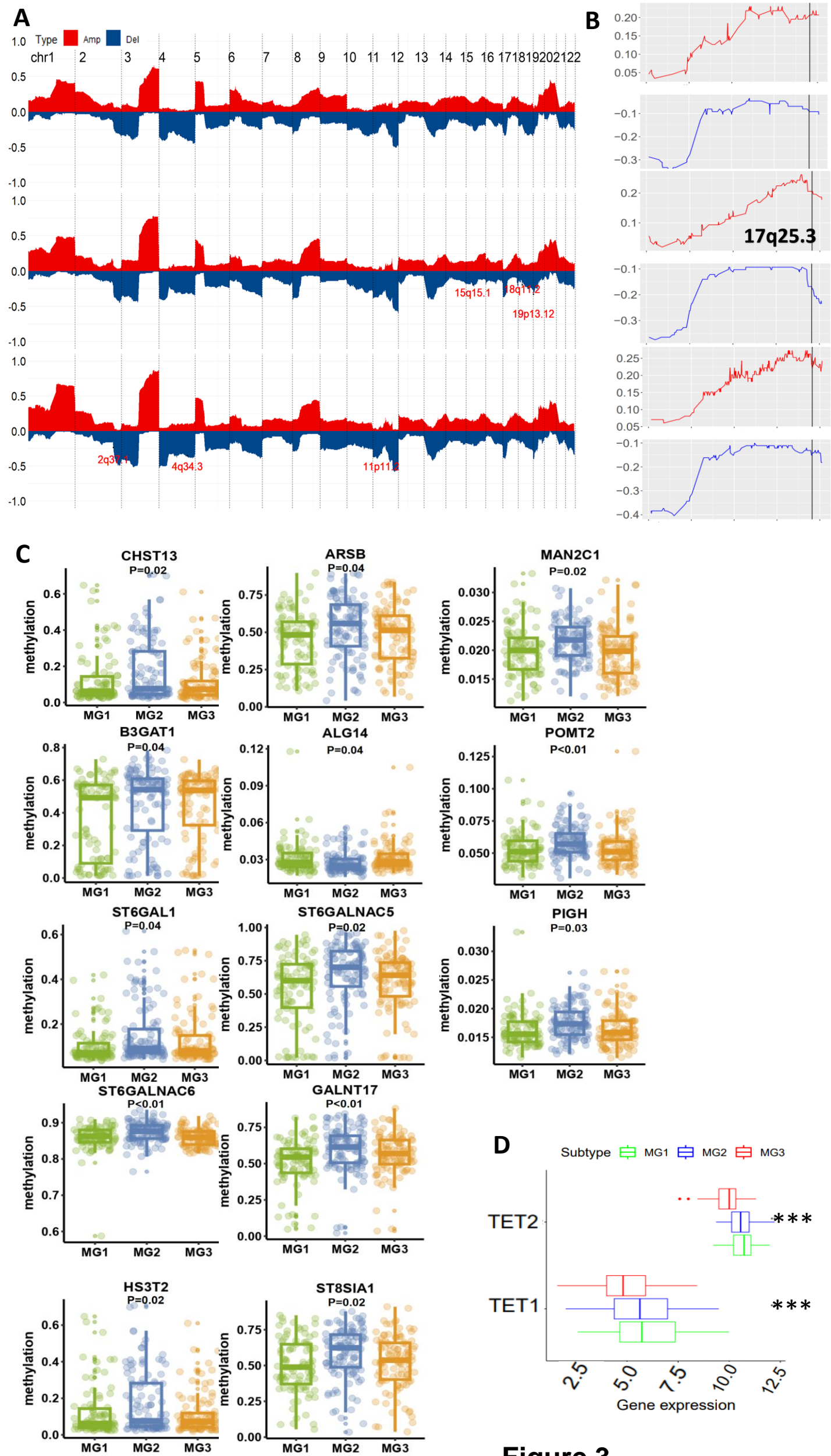

**Figure 3**

Supplement: Supplementary file 1 [file DataSheet1.zip › Supplementary_Material_Presentation/Fig 3.pdf]

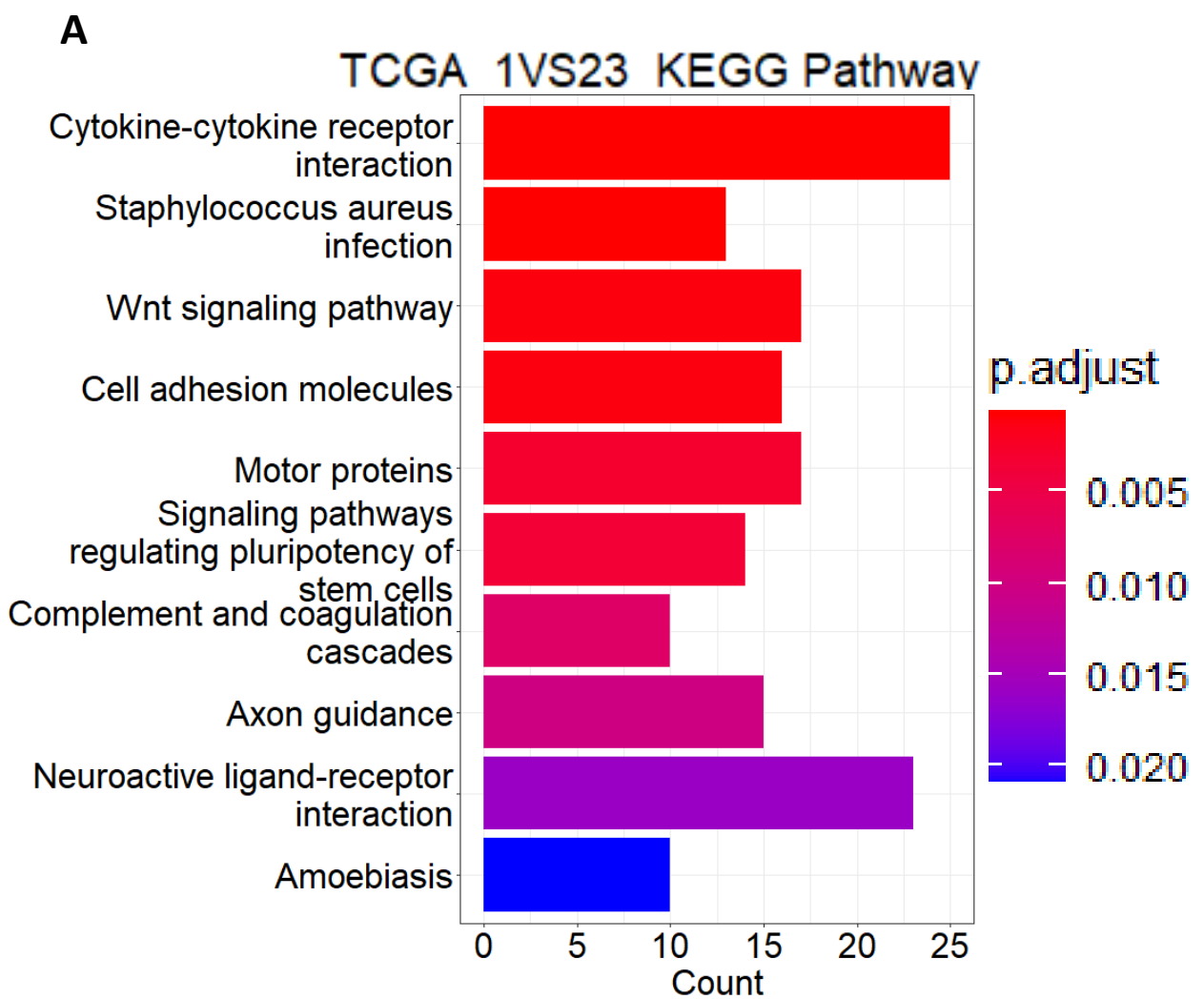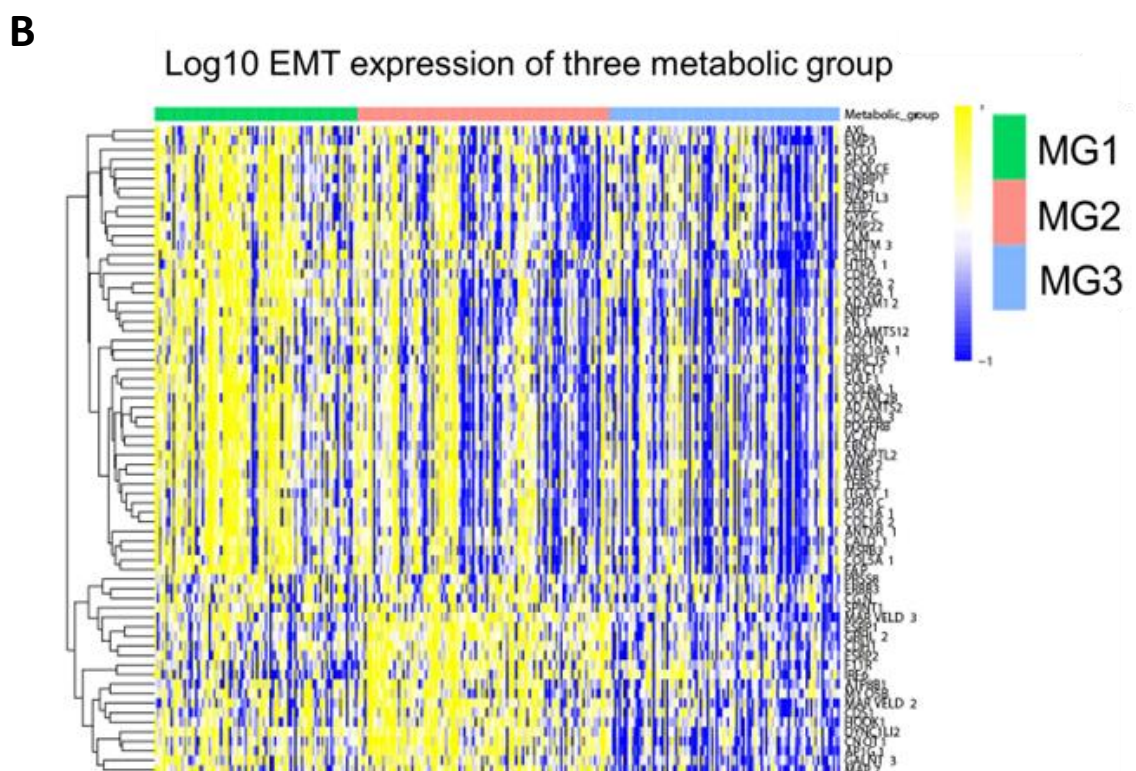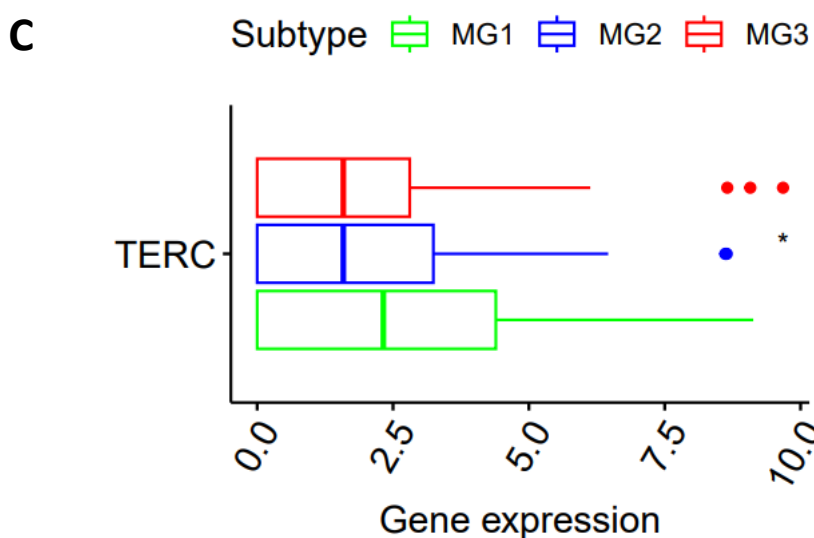

**Figure 4**

Supplement: Supplementary file 1 [file DataSheet1.zip › Supplementary_Material_Presentation/Fig 4.pdf]

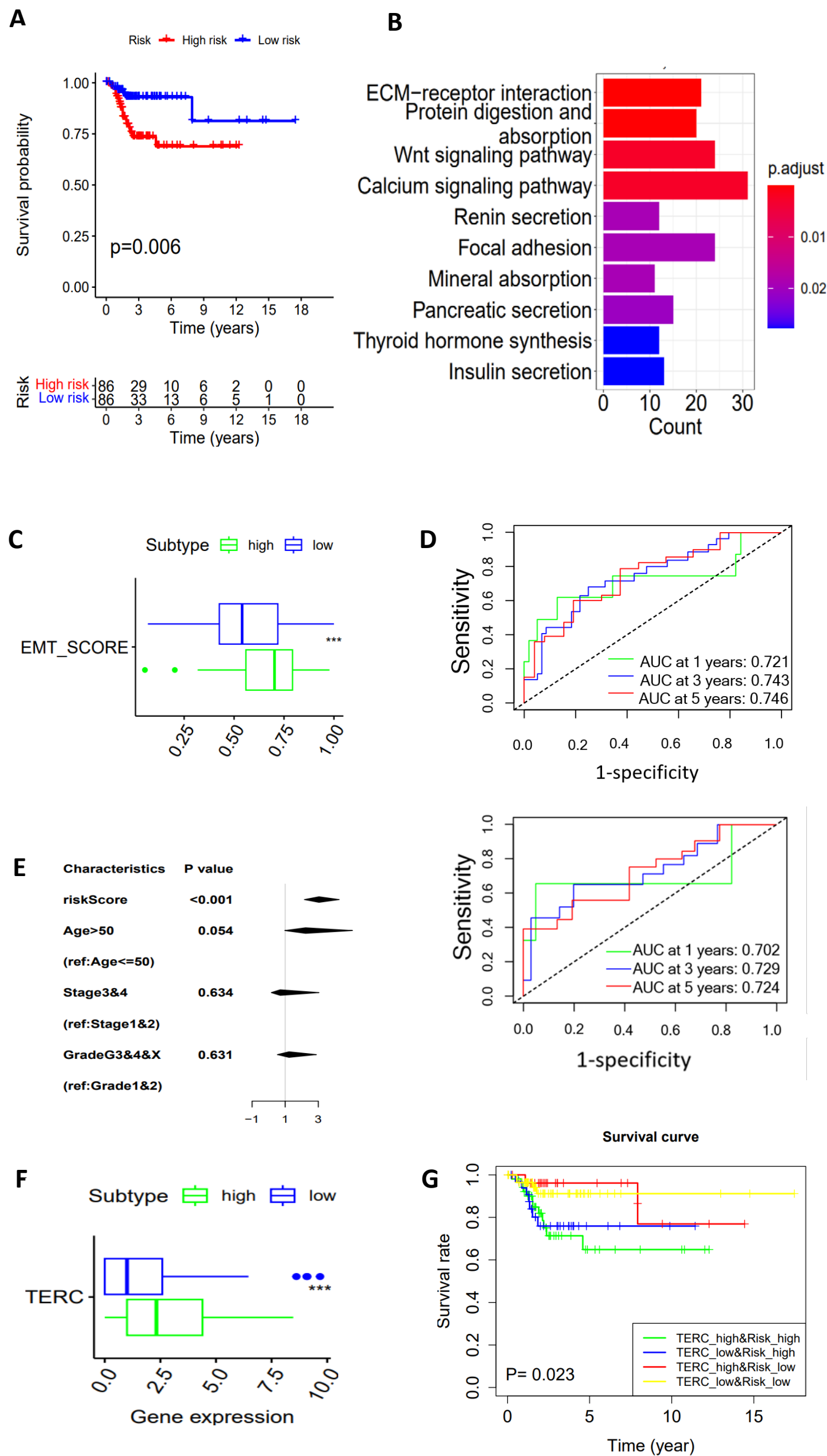

**Figure 5**

Supplement: Supplementary file 1 [file DataSheet1.zip › Supplementary_Material_Presentation/Fig 5.pdf]

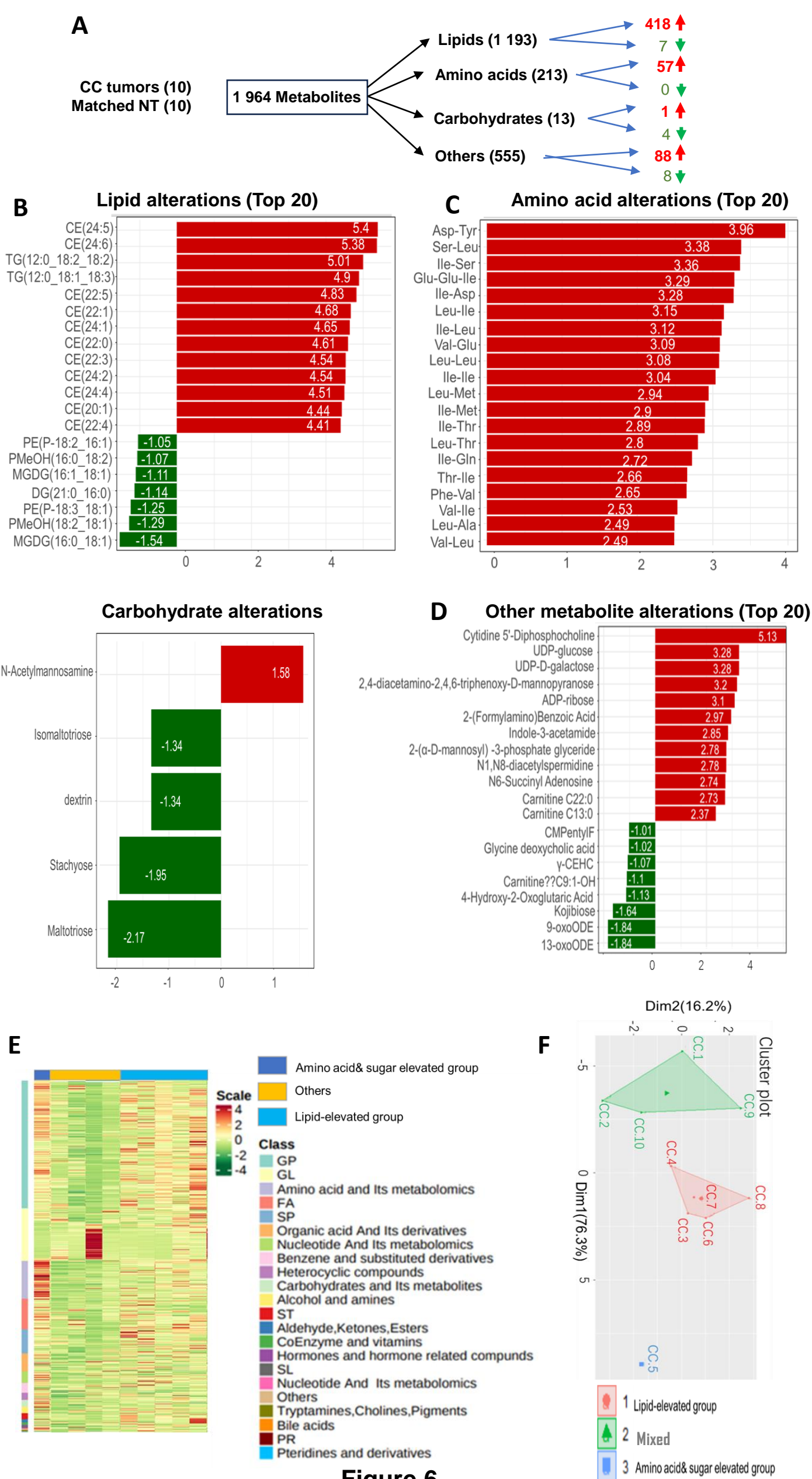

Figure 6

Supplement: Supplementary file 1 [file DataSheet1.zip › Supplementary_Material_Presentation/Fig 6.pdf]
