# Supplementary material for: Glycan dysregulation as one of major metabolic subtypes is associated with TERC overexpression and poor outcomes in cervical cancer
Source: Front Immunol. 2025 Aug 25;16:1585647. doi: 10.3389/fimmu.2025.1585647 (PMC12414962; doi:10.3389/fimmu.2025.1585647)
Supplement: Supplementary file 2 [file DataSheet2.pdf]

Figure S1. The inverse correlation between expression and DNA methylation in the key glycan genes. The TCGA CC cohort of 304 tumors were analyzed for the correlation between expression and DNA methylation in 13 key glycan genes (downloaded from CiBioportal).

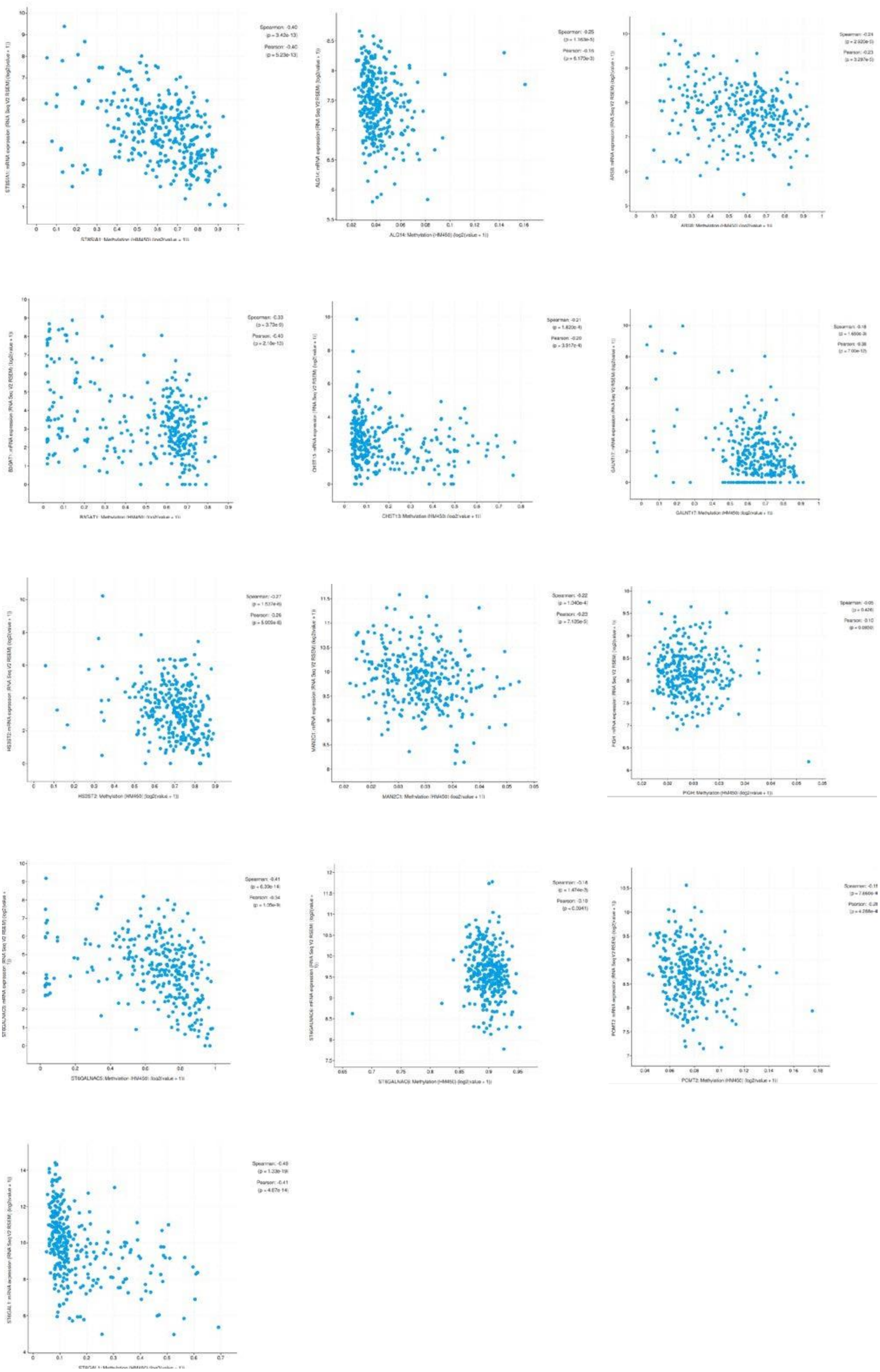

Table S1. Clinical characteristics of 10 patients with cervical cancer

	Characteristics			
	**Age(yrs)**	**HPV infection**	**Pathological diagnosis**	**Clinical stage**
**CC1/NT1**	**54**	**HPV16(+)**	**Squamous cell carcinoma**	**IIA1**
**CC2/NT2**	**47**	**HPV(-)**	**adenocarcinoma**	**IB**
**CC3/NT3**	**50**	**HPV18(+)**	**adenocarcinoma**	**IB2**
**CC4/NT4**	**59**	**HPV16(+)**	**Squamous cell carcinoma**	**IB1**
**CC5/NT5**	**57**	**HPV16(+)**	**Squamous cell carcinoma**	**IB3**
**CC6/NT6**	**50**	**HPV16/12(+)**	**Squamous cell carcinoma**	**IIB**
**CC7**	**53**	**HPV16(+)**	**Squamous cell carcinoma**	**IIA1**
**CC8/NT8**	**44**	**HPV16(+)**	**Squamous cell carcinoma**	**IA2**
**CC9**	**48**	**HPV12(+)**	**Squamous cell carcinoma**	**IB2**
**CC10**	**64**	**HPV16(+)**	**Squamous cell carcinoma**	**IB1**

Table S2 Clinical characteristics of cervical cancer patients (TCGA cohort)

Characteristics	Total Cases (%)	Metabolic Group			*P*
		MG1 (%)	MG2 (%)	MG3 (%)	
**Age at diagnosis**	304				0.062
**<=50**	186 (62)	64 (71)	62 (55)	60 (59)	
**>50**	118 (38)	26 (29)	50 (45)	42 (41)	
**HPV**	178				0.282
**Yes**	171 (96)	46 (92)	82 (91)	43 (97)	
**No**	7 (4)	4 (8)	2 (9)	1 (3)	
**Menopause status**	234				0.009
**Indeterminate**	3 (1)	1 (1)	2 (2)	0 (0)	
**Pre**	124 (53)	45 (63)	44 (50)	35 (47)	
**post**	82 (35)	15 (21)	38 (43)	29 (39)	
**Peri**	25 (11)	11 (15)	4 (5)	10 (14)	
**Tumor grade**	296				0.018
**G1**	18 (6)	6 (7)	3 (3)	9 (9)	
**G2**	135 (46)	33 (38)	46 (43)	56 (57)	
**G3**	118 (40)	39 (45)	53 (49)	26 (27)	
**G4&X**	25 (8)	9 (10)	9 (9)	7 (7)	
**Lymphovascular invasion**	150				0.003
**Yes**	79 (53)	13 (33)	38 (55)	28 (70)	
**No**	71 (47)	27 (67)	32 (45)	12 (30)	
**Nodes pathologic**	259				0.007
**N0**	133 (51)	44 (59)	53 (57)	36 (39)	
**N1**	60 (23)	10 (14)	25 (27)	25 (27)	
**NX**	66 (26)	20 (27)	15 (16)	31 (34)	
**BMI**	259				0.681
**<=18.5**	12 (5)	4 (5)	5 (5)	3 (3)	
**(18.5,24.9]**	86 (33)	23 (31)	29 (29)	34 (40)	
**(24.9,29.9]**	75 (29)	17 (23)	36 (36)	22 (26)	
**(29.9,34.9]**	43 (17)	13 (18)	19 (19)	11 (13)	
**>34.9**	43 (17)	17 (23)	10 (11)	16 (18)	
**Clinical stage**	297				0.159
**Stage**I	162 (55)	55 (62)	63 (58)	44 (45)	
**Stage**II	69 (23)	21 (24)	20 (18)	28 (29)	
**Stage**III	45 (15)	8 (9)	20 (18)	17 (17)	
**Stage**IV	21 (7)	5 (5)	7 (6)	9 (9)	
**Tabaco smoking year**	261				0.062
**<=2**	208 (80)	68 (75)	66 (72)	74 (84)	
**>2**	53 (20)	13 (25)	26 (28)	14 (16)	
**Cancer type**	304				0.004
**Adenocarcinoma**	47 (15)	24 (27)	10 (9)	13 (13)	
**Adenosquamous**	5 (2)	2 (2)	3 (3)	0 (0)	
**Squamous Carcinoma**	252 (83)	64 (71)	99 (88)	89 (87)	

Table S3 Clinical characteristics of cervical cancer patients（GSE68339）

**Characteristics**	**Total cases (%)**	**Metabolic Group**			***P***
		**MG1 (%)**	**MG2 (%)**	**MG3 (%)**	
**Clinical stage**	121				*P*>0.05
I	13(11)	3(8)	4(11)	6(13)	
II	80(66)	25(68)	23(64)	32(66)	
III&IV	28(23)	9(24)	9(25)	10(21)	
